# Carboxydotrophic growth of *Geobacter sulfurreducens*

**DOI:** 10.1007/s00253-015-7033-z

**Published:** 2015-10-19

**Authors:** Jeanine S. Geelhoed, Anne M. Henstra, Alfons J. M. Stams

**Affiliations:** Laboratory of Microbiology, Wageningen University, Dreijenplein 10, 6703 HB Wageningen, The Netherlands; NIOZ Royal Netherlands Institute for Sea Research, Korringaweg 7, 4401 NT Yerseke, The Netherlands; Centre for Biomolecular Sciences, University of Nottingham, University Park, NG7 2EF Nottingham, UK

**Keywords:** Carbon monoxide, Fumarate reduction, *Geobacter sulfurreducens*, Carboxydotrophic growth, Microbial metabolism

## Abstract

This study shows that *Geobacter sulfurreducens* grows on carbon monoxide (CO) as electron donor with fumarate as electron acceptor. *Geobacter sulfurreducens* was tolerant to high CO levels, with up to 150 kPa in the headspace tested. During growth, hydrogen was detected in very slight amounts (∼5 Pa). In assays with cell-free extract of cells grown with CO and fumarate, production of hydrogen from CO was not observed, and hydrogenase activity with benzyl viologen as electron acceptor was very low. Taken together, this suggested that CO is not utilized via hydrogen as intermediate. In the presence of CO, reduction of NADP^+^ was observed at a rate comparable to CO oxidation coupled to fumarate reduction in vivo. The *G. sulfurreducens* genome contains a single putative carbon monoxide dehydrogenase-encoding gene. The gene is part of a predicted operon also comprising a putative Fe–S cluster-binding subunit (CooF) and a FAD–NAD(P) oxidoreductase and is preceded by a putative CO-sensing transcription factor. This cluster may be involved in a novel pathway for CO oxidation, but further studies are necessary to ascertain this. Similar gene clusters are present in several other species belonging to the *Deltaproteobacteria* and *Firmicutes*, for which CO utilization is currently not known.

**Electronic supplementary material:**

The online version of this article (doi:10.1007/s00253-015-7033-z) contains supplementary material, which is available to authorized users.

## Introduction

Carbon monoxide, hydrogen, and carbon dioxide are the main constituents of synthesis gas, a so-called transportation fuel that can be produced from natural gas, coal, and refinery by-products but also by gasification of biomass or industrial and municipal solid wastes (He et al. 2009; Rauch et al. 2014). Carbon monoxide is toxic to many organisms, but a range of microorganisms from various taxonomic groups is able to use CO for growth (see reviews by Oelgeschläger and Rother 2008; Sipma et al. 2006; Sokolova et al. 2009; Techtmann et al. 2009). Carbon monoxide utilizing microorganisms may be used for conversion of CO or synthesis gas into products like hydrogen, biofuels, e.g., ethanol or butanol and other chemicals (Bengelsdorf et al. 2013).

*Geobacter sulfurreducens* is a well-known model bacterium for the study of metal and metalloid reduction and current production in a microbial fuel cell. The electron acceptors that can be used for growth are Fe(III), Mn(IV), Co(III), elemental sulfur, fumarate, malate, and electrodes of various materials, e.g., graphite, gold, and stainless steel (Afkar et al. 2005; Bond and Lovley 2003; Caccavo et al. 1994; Dumas et al. 2008; Richter et al. 2008). The initial description of the species showed a very limited electron donor utilization of only acetate and hydrogen (Caccavo et al. 1994). Later research extended the possible electron donors with formate, lactate, and electrons donated by a cathode (Call and Logan 2011; Coppi et al. 2007; Geelhoed and Stams 2011; Gregory et al. 2004; Speers and Reguera 2012).

In the genome of *G. sulfurreducens*, genes encoding the tricarboxylic acid (TCA) cycle, several different hydrogenases and genes putatively encoding formate dehydrogenase and lactate oxidase are present (Coppi 2005; Methé et al. 2003; Speers and Reguera 2012), consistent with the utilization of known electron donors. In addition, the genome contains several genes that may encode enzymes involved in the acetyl–coenzyme A pathway (Methé et al. 2003), which may have a role in acetogenic growth reactions and the assimilation of CO_2_. One of these genes putatively encodes a carbon monoxide (CO) dehydrogenase; this enzyme may be part of the acetyl–coenzyme A pathway or may be involved in the direct utilization of CO as electron donor. From previous research, no indications existed for the utilization of either of these pathways, although *G. sulfurreducens* was found to be tolerant to CO (Galushko and Schink 2000; Hussain et al. 2014).

Microbial oxidation of CO is catalyzed by carbon monoxide dehydrogenase, CO + H_2_O → CO_2_ + 2 H^+^ + 2 e^−^ (E^0′^ = −0.52 V). Many anaerobic carboxydotrophic microorganisms grow by converting CO to acetate using the acetyl — coenzyme A pathway. Some of these acetogens can grow on CO as the sole substrate for energy and carbon, e.g., *Clostridium ljungdahlii*, *Clostridium autoethanogenum*, *Moorella thermoacetica*, and *Archaeoglobus fulgidus* (Abrini et al. 1994; Daniel et al. 1990; Henstra et al. 2007; Tanner et al. 1993). To date, only few methanogens have been shown to grow on CO as sole substrate. *Methanosarcina acetivorans*, an acetotrophic methanogen, is capable of growth on CO and produces acetate, formate, methyl sulfide, and CH_4_ (Lessner et al. 2006; Moran et al. 2008; Rother and Metcalf 2004). *Methanosarcina barkeri* was observed to produce H_2_ during growth on CO but appears to grow on H_2_ as the actual electron donor (O’Brien et al. 1984). Conversely, an energy-yielding conversion of CO + H_2_O to CO_2_ + H_2_ is employed by hydrogenogenic carboxydotrophs. Examples of such organisms are phototrophs, e.g., *Rhodospirillum rubrum*, the facultative anaerobe *Citrobacter amalonaticus*, thermophilic Gram-positive bacteria like *Carboxydothermus hydrogenoformans* and *Thermosinus carboxydivorans*, and archaeal *Thermococcus* sp. (Jung et al. 1999; Kerby et al. 1995; Lee et al. 2008; Sokolova et al. 2004a, 2004b; Svetlichny et al. 1991).

Carbon monoxide may also be used as a sole electron donor for anaerobic respiratory processes with e.g., sulfate as electron acceptor. Carboxydotrophic sulfate reducers include several *Desulfotomaculum* spp., *Desulfovibrio vulgaris* (strain Madison), *Desulfovibrio desulfuricans*, *Desulfosporosinus orientis*, and *Archaeoglobus fulgidus* (Davidova et al. 1994; Henstra et al. 2007; Klemps et al. 1985; Lupton et al. 1984; Parshina et al. 2005). Carbon monoxide conversion may also be coupled to the reduction of e.g., fumarate, AQDS (by *C. hydrogenoformans* and *C. ferrireducens*; Henstra and Stams 2004), elemental sulfur, thiosulfate, and DMSO (by *Sulfurospirillum carboxydovorans*; Jensen and Finster 2005). Many of the respiratory CO oxidizers produce hydrogen that may function as the true electron donor for respiration (Sipma et al. 2006).

Here, we show that *G. sulfurreducens* grows with CO as electron donor coupled to fumarate reduction. During growth, only very slight concentrations of hydrogen were observed. Together with a very low hydrogenase activity in cell-free extracts of CO-grown cells, this suggests that CO was utilized directly, i.e., without conversion of CO to hydrogen. Genome analysis showed that *G. sulfurreducens* contains one anaerobic monofunctional carbon monoxide dehydrogenase-encoding gene, present in a predicted operon with a possible function in respiratory metabolism. We detected similar gene clusters in other *Deltaproteobacteria* and in members of the class *Clostridia*, which may indicate a wider potential for CO utilization than what is currently known.

## Materials and methods

### Growth experiments

*Geobacter sulfurreducens* strain PCA (DSM 12127^T^) was obtained from the German Collection of Microorganisms and Cell Cultures (DSMZ, Braunschweig, Germany). Cultures were grown at 35 °C in closed bottles with anaerobic bicarbonate buffered medium, containing (mM) NaCl 5.1, MgCl_2_ 0.5, CaCl_2_ 0.75, NH_4_Cl 5.6, Na_2_SO_4_ 0.35, KH_2_PO_4_ 3, Na_2_HPO_4_ 3, NaHCO_3_ 50, supplemented with trace elements (1 ml l^−1^, (Newman et al. 1997)), Se/W solution (1 ml l^−1^, (Widdel and Bak 1992)), vitamins (2 ml l^−1^, (Wolin et al. 1963)), and resazurin (0.5 mg l^−1^). The medium was reduced with 0.5 mM Na_2_S. A liquid volume of 45 ml was used in 125-ml bottles. Larger volumes, 190 ml liquid in 570-ml bottles, were cultured for preparation of cell-free extracts. The headspace contained N_2_, CO_2_, and CO at the indicated pressures. *Geobacter sulfurreducens* was grown with 40 mM formate, 0.8 mM acetate and 40 mM fumarate, and N_2_/CO_2_ (140:20 kPa) in the headspace until the substrate was depleted and subsequently transferred (10 % *v*/*v*) to medium with 40 mM fumarate and N_2_/CO_2_/CO (100:20:40 kPa) in the headspace. Subsequent transfers for growth on CO were made with 10 % (*v*/*v*) into fresh medium. Cultures were also grown at a range of initial CO concentrations (up to 150 kPa) by replacing N_2_ in the headspace with CO.

Carbon monoxide and H_2_ were analyzed by gas chromatography (Hitachi GC 14B equipped with a thermal conductivity detector and molecular sieve column). Organic acids were separated by liquid chromatography on a Varian Metacarb 67H 300 mm column and quantified using refractive index or by absorption at 210 nm. Cell pellets from centrifuged culture samples or from cell-free extracts were analyzed for protein content according to Lowry et al. (1951). Growth yields were calculated assuming a protein content of 50 % and an average biomass composition of CH_1.8_O_0.5_N_0.2_. The yield on acetate + fumarate was calculated from the pre-growth phase of the experiment shown in Fig. 2 in which 14 mM of acetate was used.

### Enzymatic activity measurements

Cell-free extracts were prepared from cells grown with 40 kPa initial CO concentration, or 16 mM acetate, or 40 mM formate + 0.8 mM acetate. Fumarate (40 mM) was used as electron acceptor for all the cultures. After at least three or four transfers on the respective substrate, cells were grown to mid-to-end log phase. Cell-free extract preparation was carried out in anoxic conditions in a glove box with a gas phase of N_2_/H_2_ (96:4 kPa), except centrifugation which was carried out in airtight closed tubes. Cells were collected by centrifugation at 4 °C and washed in 0.1 M KP_i_ buffer (pH 7.2) supplemented with 2.5 mM MgCl_2_ and 2.5 mM dithiothreitol. The pellet was resuspended in 1 ml of the buffer and disrupted by sonication. Remaining cells and cell debris were removed by centrifugation at 13,000 rpm for 10 min in an Eppendorf centrifuge. The prepared cell-free extract was kept in a N_2_ gas phase and used for activity assays the same day.

Enzyme activity in cell-free extracts was assayed spectrophotometrically in stoppered N_2_-flushed cuvettes at 35 °C with 50 mM anoxic Tris–HCl (1 ml, pH 8) as buffer. Reducing conditions were created by addition of sodium dithionite (<0.2 mM). Carbon monoxide dehydrogenase, hydrogenase, formate dehydrogenase, NADH oxidase, and NADPH oxidase activities were assayed with 2 mM benzyl viologen as electron acceptor. The activities were calculated from the increase in absorbance after the addition of cell-free extract (578 nm; ε_BV_ = 9.78 M^−1^ cm^−1^). Sodium formate (20 mM final concentration), NADH (5 and 20 mM), and NADPH (5 mM) were added to N_2_-saturated buffer. Carbon monoxide and hydrogen were supplied as gas-saturated buffer. Production of NADH and NADPH in the presence and absence of CO was measured at 340 nm (ε_NADH,NADPH_ = 6.23 M^−1^ cm^−1^). NAD^+^ or NADP^+^ was added at a final concentration of 5 or 20 mM to CO or N_2_-saturated buffer. The effect of the addition of 2 mM FAD on NAD(P)H production was also tested.

Hydrogen evolution activity was assayed using 35-ml closed bottles containing 5 ml of anoxic buffer. Electrons for hydrogen production were supplied from reduced methyl viologen, using 4 mM methyl viologen and 20 mM sodium dithionite in the buffer, or from CO supplied as CO/N_2_ (50:100 kPa) in the headspace. The assay was started by addition of cell-free extract (10–25 μl), and hydrogen production was measured in 0.5 ml headspace gas samples taken in time over a period of several hours.

### CODH gene cluster analysis

The gene putatively coding for carbon monoxide dehydrogenase (CODH) was listed in the paper describing the genome of *G. sulfurreducens* (Methé et al. 2003). The characteristics of CODH were studied using comparative analysis with CODH(−ACS) from other CO-utilizing organisms. The CODH gene neighborhood was studied using IMG (img.jgi.doe.gov; Markowitz et al. 2014) and BlastP (http://blast.ncbi.nlm.nih.gov; Altschul et al. 1990) searches against proteins of known CO-utilizing organisms. Operon prediction was provided by the Prokaryotic Operon Database (http://operons.ibt.unam.mx/OperonPredictor/; Taboada et al. 2012). Gene clusters related to the CODH cluster in *G. sulfurreducens* were investigated using BlastP, by comparison of neighborhood regions in IMG, and using the Neighborhood option in SMART (smart.embl-heidelberg.de; Letunic et al. 2015; Schultz et al. 1998). Protein sequences of putative CooS subunits were downloaded from IMG or UniProt (http://www.uniprot.org) and aligned using MUSCLE (Edgar 2004). A phylogenetic tree of CooS sequences was constructed using the neighbor-joining method with *p* distance model in MEGA5 (Tamura et al. 2011). Bootstrap support was calculated for 100 replicates.

## Results

### Carbon monoxide as electron donor for growth

**Fig. 1:**
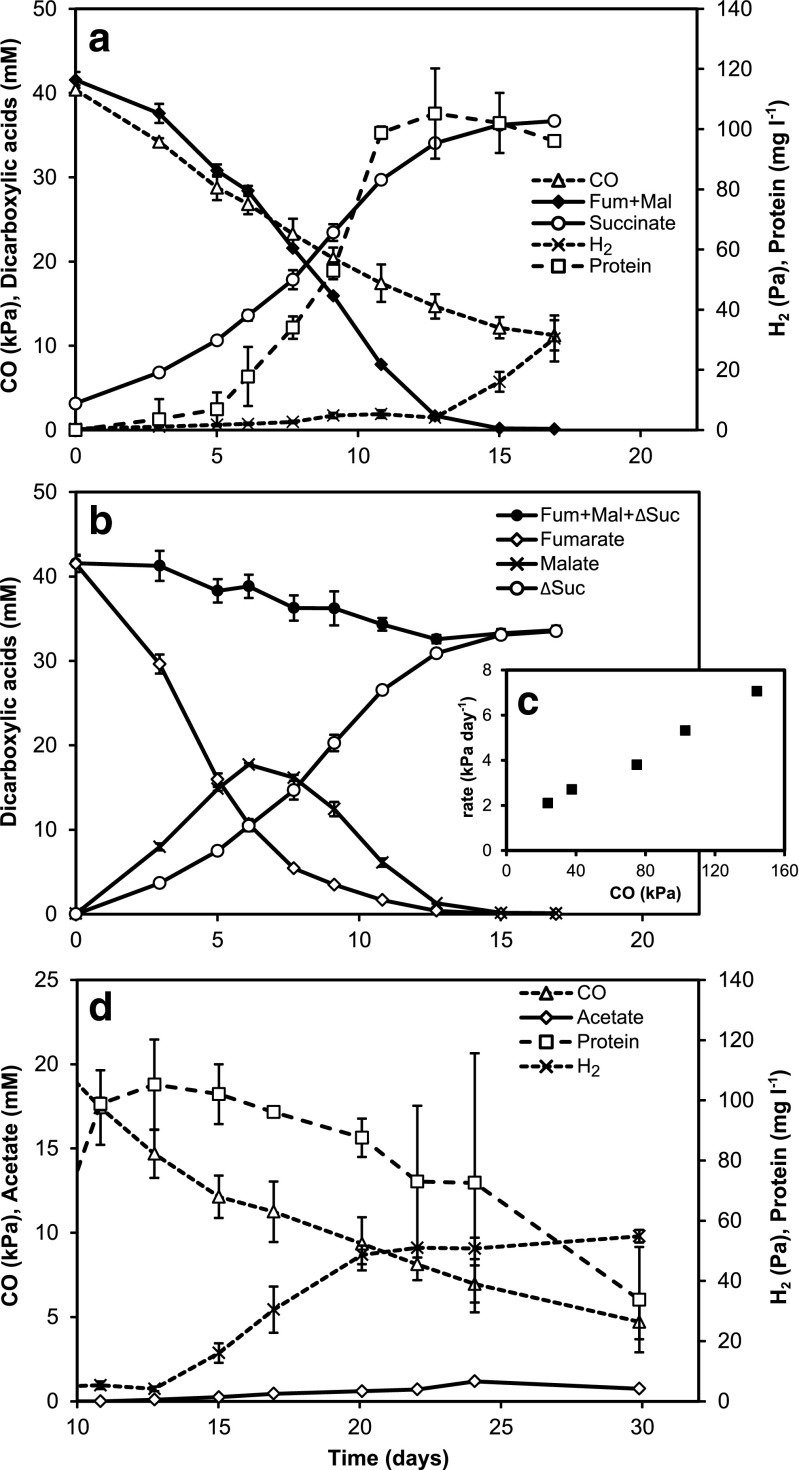
Growth of *Geobacter sulfurreducens* with CO and fumarate. **a** The oxidation of CO is coupled to the reduction of fumarate to succinate, resulting in an increase in cell protein in the culture liquid. Slight amounts of H_2_ are also produced. Data points are averages (±SD) for two cultures. **b** Fumarate is partially hydrolyzed to malate, which accumulates transiently. Over the course of the experiment, a decrease in total dicarboxylic acid concentration in the culture liquid was observed. ΔSuc, succinate produced. **c** *G. sulfurreducens* tolerates high concentrations of CO. The observed rate of CO oxidation increased with larger CO pressure in the headspace. **d** Uptake of CO was observed in the absence of fumarate. Small amounts of H_2_ and acetate were produced, but the protein concentration in the culture decreased strongly

The use of carbon monoxide as energy source for growth of *G. sulfurreducens* was tested with fumarate as electron acceptor. The inoculum was grown with 40 mM formate + 0.8 mM acetate and 40 mM fumarate until both formate and fumarate were exhausted and then transferred (10 % *v*/*v*) to fresh medium with 40 kPa CO in the headspace and 40 mM fumarate as electron acceptor. *Geobacter sulfurreducens* oxidized carbon monoxide coupled to the reduction of fumarate to succinate, which yielded energy for growth (Fig. 1a). Approximately 0.8 mmol CO was converted in 15 days, which resulted in a biomass production of approximately 100 mg protein l^−1^. During growth, very slight amounts of hydrogen, in the range of 5 Pa, were observed.

**Fig. 2:**
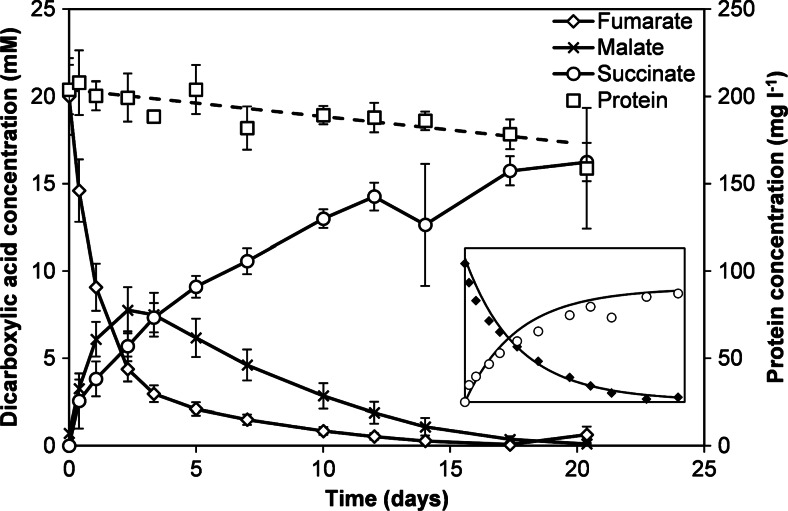
*Geobacter sulfurreducens* cells metabolize fumarate to succinate. In the absence of an added electron donor, fumarate conversion to succinate occurred in a ratio ∼1:0.8 and did not yield energy for growth. Transient production of malate was observed. Cells were pre-grown with acetate as electron donor which was depleted at *t* = 0. [Fumarate + malate] consumption and succinate production could be described with an exponentional function using *k* = 1 (g protein l^−1^)^−1^ day^−1^ (inset)

Part of the supplied fumarate was hydrolyzed to malate resulting in transient malate accumulation (Fig. 1b). After all fumarate and malate were converted, growth ceased. Less than stoichiometric amounts of succinate were produced compared to the sum of fumarate and malate consumed. In part, this may be explained by the utilization of organic carbon as C-source for biomass production which required approximately 80 μmol fumarate that is equivalent to 2 mM for the cultures in Fig. 1. In addition, in cultures without CO, we observed that fumarate was metabolized to succinate in a process that did not result in growth (Fig. 2). Production of malate as intermediate was observed. The overall conversion occurred in a ratio of approximately 1 fumarate:0.8 succinate. Conversion of 20 mM fumarate took 20 days, and during this time, the biomass concentration decreased by approximately 0.75 % per day (*R*^2^ = 0.40). The consumption of [fumarate + malate] fitted an exponential function with *k* = 1 (g protein l^−1^)^−1^ day^−1^ (Fig. 2 inset). With the derived rate constant, we calculated the turnover of [fumarate + malate] to succinate in the growth experiment with CO (Fig. 1). This resulted in an estimated amount of 200 μmol [fumarate + malate] that was converted, equivalent to a concentration of 5 mM in the culture liquid. The decrease in the observed concentrations of [fumarate + malate + produced succinate] can therefore be explained from the use of organic C as C-source and fumarate metabolism (Fig. 1b).

In the growth experiment, the CO pressure in the headspace decreased linearly over time (Fig. 1a), and also, [fumarate + malate] consumption and succinate production changed approximately linearly. Biomass protein concentrations measured in the first part of the experiment may be underestimated because of some initial biomass deposition on the bottom of the flask. The CO uptake rate was not affected by the biomass density, suggesting that transfer of CO over the gas–liquid interface was limiting CO uptake and therefore growth. Indeed, in separate batch experiments, an approximately linear correlation between CO concentration in the headspace and the rate of CO oxidation was observed (Fig. 1c). Agitation of the incubated bottles to improve the transfer of CO into the aqueous phase, however, resulted in impaired CO oxidation activity. This may suggest that small cell aggregates were required to prevent CO toxicity or to maintain well-reduced conditions for actively metabolizing cells. The CO concentration in the liquid in equilibrium with a pressure of 150 kPa CO is approximately 1.3 mM (Lide and Frederikse 1995).

After the electron acceptors fumarate and malate were depleted (Fig. 1a, day 15 of the growth experiment), conversion of CO by *G. sulfurreducens* continued at a slower rate. CO conversion led to an increase in the H_2_ concentration to around 50 Pa and the production of approximately 1.2 mM acetate (∼5 μmol acetate in total, day 15–day 30, Fig. 1d). In this time period, approx. 0.2 mmol CO was taken up. Concomitantly, the biomass concentration decreased strongly with about 70 mg protein l^−1^, or around 0.23 mmol C-biomass.

### Gene cluster with a cytoplasmic monofunctional CODH

The genome of *G. sulfurreducens* contains one gene putatively coding for CODH (GSU2098; Methé et al. 2003). Other genes in close vicinity show homology to other subunits of CODH complexes. All proteins encoded by genes in this cluster are predicted to be cytoplasmic (Gardy et al. 2005). GSU2099 putatively encodes a CO-sensing bacterial transcriptional regulator (RcoM, 232 aa), that shows 27 % amino acid identity (48 % positives) to RcoM of *Burkholderia xenovorans*. RcoM is a single-component transcription factor that regulates expression of the aerobic (*cox*) and likely also the anaerobic (*coo*) CO-oxidation systems and is fundamentally different from the CooA regulators (Kerby et al. 2008; Marvin et al. 2008). RcoM joins a PAS domain containing a CO-binding heme and a LytTR DNA-binding domain. In *Rhodospirillum rubrum*, both RcoM and CooA are present (Kerby et al. 2008), but in *G. sulfurreducens*, only RcoM is present and precedes genes involved in CO oxidation, suggesting their expression is controlled by RcoM.

GSU2098 encodes a protein (640 aa) with homology to CooS, the catalytic subunit of anaerobic CODH. CooS of *G. sulfurreducens* contains conserved cysteine residues that are ligands to the D- and B-[4Fe–4S] clusters and to [3Fe–4S] and Ni of the C-cluster (Lindahl 2002). The catalytic subunit may be part of distinct CODH complexes with different physiological roles, which is exemplified by the presence of five homologs in *C. hydrogenoformans* (Wu et al. 2005). The *cooS* gene in *G. sulfurreducens* is distinct from genes that encode bifunctional CODH/acetyl CoA synthase (CODH/ACS), and also, genes that encode other subunits of the acetyl-CoA complex are lacking, suggesting it encodes a monofunctional CODH.

GSU2097 putatively encodes a homolog of CooC, an accessory protein (284 aa) involved in ATP-dependent Ni-insertion into CODH (Jeon et al. 2001). CooC contains a ATP/GTP-binding P-loop (Kerby et al. 1997) that is conserved in *G. sulfurreducens* CooC. In *Rhodospirillum rubrum*, expression of *cooCTJ* is required for in vivo insertion of Ni into CooS upon exposure to CO, and mutants lacking these genes required increased Ni concentrations to allow CO-dependent growth (Kerby et al. 1997; Watt and Ludden 1999). BlastP searches of CooT and CooJ sequences against the genome of *G. sulfurreducens* did not result in significant hits. However, also in *Carboxydothermus hydrogenoformans*, no homologs of *cooTJ* are present, indicating it is not essential for CO utilization.

GSU2096 encodes an iron–sulfur cluster-binding protein (197 aa), containing binding sites for two [4Fe–4S] clusters. The protein shares 40 % amino acid sequence identity with CooF2 (CHY0735) and 32 % with CooF1 (CHY0086) of *C. hydrogenoformans*. CooF is proposed to mediate electron transfer from CooS via the Fe–S clusters (Singer et al. 2006).

GSU2095 (422 aa) has been annotated as an NADH oxidase (Methé et al. 2003). NADH oxidases catalyze the oxidation of NADH with oxygen to hydrogen peroxide or water and therefore may contribute to antioxidant activity in anaerobic bacteria. In addition, NADH oxidases may also pass on electrons to other electron acceptors and be involved in respiratory pathways (Kengen et al. 2003; Reed et al. 2001; Yang and Ma 2007). The sequence contains FAD and NAD(P)-binding motifs (Geueke et al. 2003; Scrutton et al. 1990; Yang and Ma 2007). A homolog of this FAD-NAD(P) oxidoreductase (FNOR) with 29 % amino acid sequence identity is present in *C. hydrogenoformans* in an operon (CHY0735–0738) with a CODH catalytic subunit (CooSIV), an Fe–S cluster-binding protein (CooF), and a rubrerythrin-like protein. The proposed function of this FNOR is to act as electron carrier from oxidized CO to rubrerythrin for the detoxification of reactive oxygen species (Wu et al. 2005).

Considering the short intergenic distances of the genes in the cluster GSU2098–2095 and predicted protein interactions evaluated with STRING (Jensen et al. 2009), the genes GSU2098–2095 are predicted to constitute an operon (Taboada et al. 2010) and hence to be co-expressed. The predicted function of three of the genes in the operon (*CooS*, *CooC*, and *CooF*) strongly suggests that the operon is involved in CO metabolism and is expressed under the control of the single-component RcoM-type transcription factor (GSU2099). Therefore, in *G. sulfurreducens*, a respiratory pathway of electrons from CO may be hypothesized from the active site in CooS (GSU2098) via the Fe–S clusters of CooF (GSU 2096) and the FNOR (GSU2095) onto the electron carriers NAD^+^ or NADP^+^. The electrons may subsequently be delivered to the quinone pool in the membrane and used for the reduction of fumarate.

### Enzyme activity measurements

**Fig. 3:**
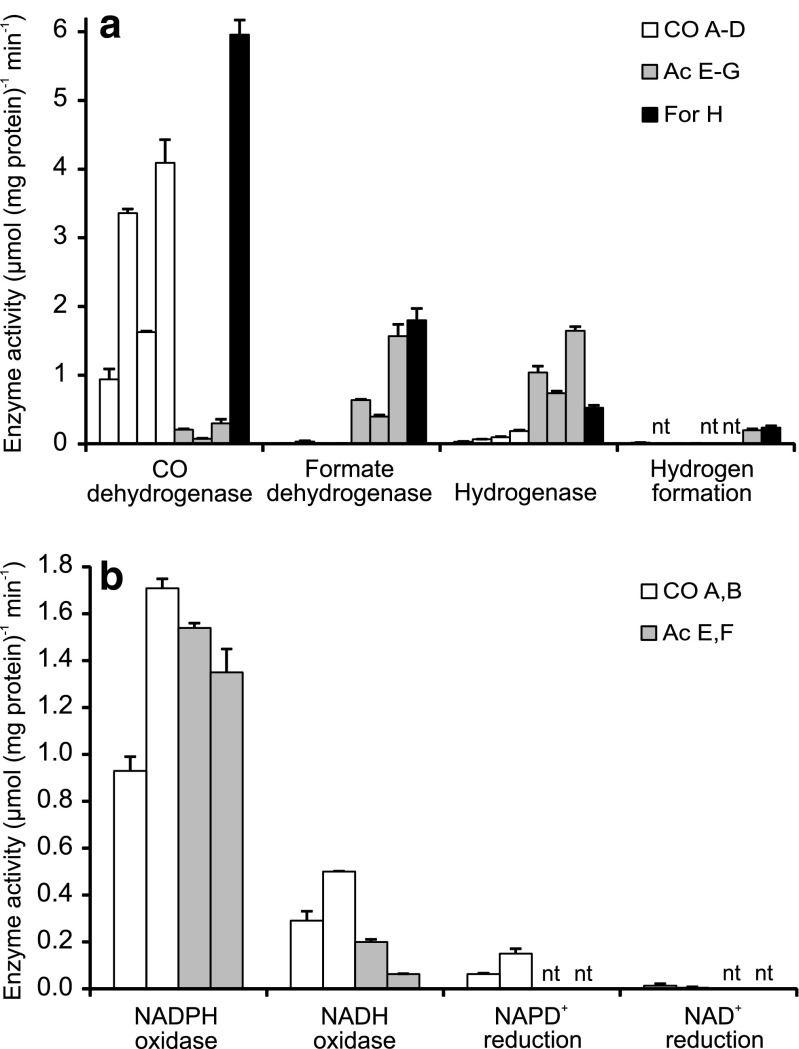
Specific enzyme activities of cell-free extracts of cultures grown with CO, acetate, or formate. **a** Carbon monoxide dehydrogenase, formate dehydrogenase, and hydrogenase activity measured as hydrogen consumption and as hydrogen production. **b** NADPH and NADH oxidase activity and CO-dependent reduction of NADP^+^ and NAD^+^. Bars with the same color indicate the activities (average ± SD) for multiple cell-free extract preparations of cultures grown with the same electron donor, see Table S1

Cell-free extract was prepared from cells cultured at the indicated conditions for at least three transfers (10 % inoculum). Specific activity of CO dehydrogenase, formate dehydrogenase, hydrogenase, and hydrogen evolution was measured in cell-free extracts (CFE, multiple preparations) from cultures grown with CO, acetate, and formate as electron donors and with benzyl viologen as artificial electron acceptor (Fig. 3; Table S1). Cultures grown with formate had high CO dehydrogenase activity and were used as inoculum for testing growth on CO. In cell-free extract preparations of cells grown with CO (CFE A–D), the CO dehydrogenase activity ranged from 0.9 to 4.1 μmol CO mg protein^−1^ min^−1^, and of cells grown with acetate (CFE E–G), it ranged from 0.08 to 0.3 μmol mg protein^−1^ min^−1^. Activity of formate dehydrogenase and hydrogenase was virtually absent in CO-grown cells, whereas relatively high levels of activity ranging from 0.4 to 1.7 μmol formate or H_2_ mg protein^−1^ min^−1^ were present in acetate-grown cells. Hydrogenase activity assayed in the reverse direction, i.e., hydrogen production using electrons donated by dithionate via methyl viologen, was also virtually absent in CO-grown cells and amounted to about 0.2 μmol H_2_ mg protein^−1^ min^−1^ in acetate- and formate-grown cells (CFE G and H, Fig. 3a). Moreover, formation of hydrogen in the presence of CO was not observed in incubations with cell-free extracts of CO-grown cells (tested for CFE A, C, and D).

NADH oxidase and NADPH oxidase activity was assayed with benzyl viologen as artificial electron acceptor. This activity is in the direction opposite to what we hypothesized for cells growing with CO. The observed specific NADPH oxidase activity of 0.9 and 1.7 μmol mg protein^−1^ min^−1^ in CO-grown cells (CFE A and B, Fig. 3b) was in the same order of magnitude as the CO dehydrogenase activity. The activity of NADH oxidase was about three-fold lower compared to NADPH oxidase activity, and an increase in the NADH concentration from 5 to 20 mM did not have much effect on the oxidation rate (Table S1). In acetate-grown cells, activity of NADPH oxidase was in the same range, and NADH oxidase activity was somewhat lower compared to CO-grown cells.

In the presence of CO, NADPH was produced at a rate of 0.06–0.15 μmol mg protein^−1^ min^−1^. This rate is comparable to the rate of CO consumption in the growth experiment, which was 0.048 μmol mg protein^−1^ min^−1^ calculated for the early phase of the growth curve (*t* = 6.1 days) to reduce the impact of limiting CO transfer on the calculated consumption rate. Compared to enzyme activities measured with the very strong artificial electron acceptor benzyl viologen, the NADP^+^ reduction rate in the presence of CO amounted to 6.7–8.8 % of the NADPH oxidase activity and 4.5–6.6 % of CO dehydrogenase activity. The NAD^+^-reducing activity was lower, in agreement with the observed lower NADH oxidase activity compared to NADPH oxidase activity. Addition of FAD (2 mM) had no effect on the rate of NAD(P)^+^ reduction. In the absence of CO, NAD(P)H was not produced (Fig. 3b; Table S2). The enzyme activity measurements with CO-grown *G. sulfurreducens* cells show that the oxidation of CO does not result in production of H_2_, but that electrons from CO can be transferred to NADP^+^.

### Similar *cooS*-containing gene clusters in other bacteria

**Fig. 4:**
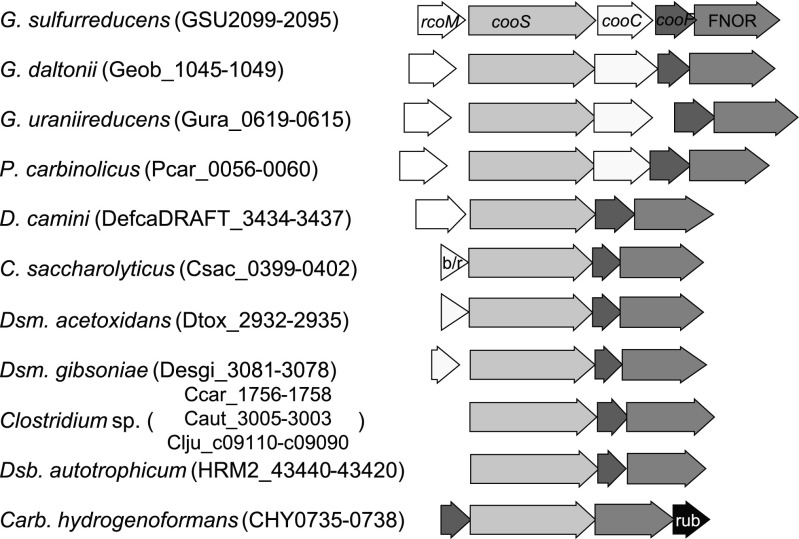
Carbon monoxide dehydrogenase gene cluster in *Geobacter sulfurreducens* and similar gene clusters present in other organisms. Putatively encoded gene functions: CO-sensing transcriptional regulator (*rcoM*); CO dehydrogenase catalytic subunit (*cooS*); accessory protein (*cooC*); Fe–S cluster - binding protein (*cooF*); FAD–NAD oxidoreductase (FNOR); BadM/Rrf2 type transcriptional regulator (b/r); rubrerythrin (rub). *Clostridium* sp. denotes *Cl. carboxidivorans*, *Cl. autoethanogenum*, and *Cl. ljungdahlii*

In the genomes of close relatives of *G. sulfurreducens*, *Geobacter uraniireducens*, *Geobacter daltonii*, and *Pelobacter carbinolicus*, gene clusters that are comparable in composition and gene order to the CODH cluster in *G. sulfurreducens* are present (Fig. 4). The amino acid sequence identities of the encoded putative proteins compared to *G. sulfurreducens* are CooS >75 %, CooC >60 %, CooF >66 %, and FNOR >61 %. The putative RcoM transcriptional regulator showed >62 % amino acid sequence identity for the *Geobacter* species, and 39 % identity (65 % positives) for the putative protein in *Pelobacter carbinolicus*. Another highly similar gene cluster, but lacking a gene coding for CooC, is present in *Deferrisoma camini*, a moderately thermophilic iron(III)-reducing bacterium also belonging to *Deltaproteobacteria* (Slobodkina et al. 2012).

Gene clusters consisting of sequential genes putatively coding for CooS, CooF, and FNOR are present in the sulfate reducers *Desulfobacterium autotrophicum*, *Desulfotomaculum acetoxidans*, and *Desulfotomaculum gibsoniae*, and in the thermophilic fermenting bacterium *Caldicellulosiruptor saccharolyticus*. None of these organisms is known to be able to use CO in respiratory metabolism (Table S2). In the presently known anaerobic CO utilizing microorganisms, gene clusters most similar to GSU2099–2095 are found in the *Clostridium* species *Clostridium carboxidivorans*, *Cl. autoethanogenum*, and *Cl. ljungdahlii* that contain very similar CooS–CooF–FNOR gene clusters (Fig. 4) (Bruno-Barcena et al. 2013; Köpke et al. 2010; Paul et al. 2010). *Carboxydothermus hydrogenoformans* cluster CHY0735–0738 genes putatively code for CooF–CooS(IV)–FNOR and rubrerythrin (Wu et al. 2005).

Phylogenetic analysis of monofunctional CODH (CooS) protein sequences showed that CooS sequences of *Geobacter*/*Pelobacter* spp. and *Deferrisoma camini* are placed together (Group I in Fig. S1). The proteins in Group I are most closely related to CooS proteins of the Archaeal *Thermococcus* spp. that oxidize CO + H_2_O to H_2_ + CO_2_ (Lee et al. 2008; Sokolova et al. 2004b). In contrast, the deltaproteobacterial CooS proteins of the CO-oxidizing sulfate-reducing bacterium *Desulfovibrio desulfuricans* are more distantly related. The CooS protein sequences of *Clostridium carboxidivorans*, *Cl. autoethanogenum*, and *Cl. ljungdahlii* that are part of CooS–CooF–FNOR gene clusters grouped together with the CooS protein sequences of *Caldicellulosiruptor saccharolyticus* and *Desulfotomaculum acetoxidans* and *Dsm. gibsoniae* that are also part of such clusters (Group II, Fig. S1).

## Discussion

This study provides the first evidence for the use of CO as electron donor for anaerobic respiration coupled to growth by *G. sulfurreducens*. Carbon monoxide was used with fumarate as electron acceptor. The growth rate increased with higher CO concentrations and was tested up to 150 kPa in the headspace (Fig. 1). However, mass transfer of CO over the gas–liquid interface was limiting growth. In the absence of the electron acceptor fumarate, growth did not occur.

The ability to use CO as energy source among Proteobacteria is limited. The only other known deltaproteobacterial CO oxidizers are *Desulfovibrio vulgaris* (strain Madison) and *Desulfovibrio desulfuricans*, which have a limited CO tolerance of up to 20 kPa. Other CO-converting Proteobacteria include anaerobic phototrophs, e.g., *Rhodospirillum rubrum,* sulfur-reducing *Sulfurospirillum carboxydovorans*, hydrogenogenic *Citrobacter amalonaticus* (Jensen and Finster 2005; Jung et al. 1999; Kerby et al. 1995), and aerobic species (see review by Meyer et al. 1990).

Many CO-utilizing microorganisms possess the acetyl–coenzyme A pathway and are able to use CO also as C-source. In contrast, the genome of *G. sulfurreducens* encodes only one monofunctional CODH and no genes putatively encoding CODH/ACS which is an important component of the acetyl–coenzyme A pathway. As C-source for growth, fumarate was probably used, as was also shown for ^13^C-fumarate labeling studies of acetate + fumarate grown cells (Yang et al. 2010).

Conversion of CO + H_2_O to H_2_ + CO_2_ and subsequent utilization of H_2_ as electron donor in a respiratory pathway is frequently observed in carboxydotrophic anaerobic respiration. The *G. sulfurreducens* genome contains genes encoding two membrane-bound periplasmic hydrogenases and one cytoplasmic hydrogenase (Coppi 2005; Coppi et al. 2004). A second previously suggested cytoplasmic hydrogenase has recently been proposed to be part of an electron-bifurcating NADH dehydrogenase/heterodisulfide reductase (Coppi 2005; Ramos et al. 2015). In cultures with CO + fumarate, very small concentrations of H_2_, up to 5 Pa, were observed in the headspace (Fig. 1). Although this could hint to immediate and almost complete utilization of any produced H_2_, hydrogenase activity (assayed both as H_2_ oxidation and H_2_ evolution) in cell-free extracts of *G. sulfurreducens* grown with CO was low. Hydrogen production in incubations of cell-free extracts with CO was not observed (Fig. 3, Table S1), making it unlikely that CO utilization occurs via hydrogen as intermediate.

The only putative CODH present in the genome is encoded in a predicted operon with genes putatively coding for proteins known to be involved in CO metabolism (accessory protein CooC and electron transfer subunit CooF) and with a FAD-dependent NAD(P) oxidoreductase. The operon is preceded by a putative CO transcriptional regulator RcoM. The observed CO-dependent production of NADPH is an indication that the operon may constitute a novel CO respiratory pathway for direct utilization of CO without production of H_2_ as intermediate. However, more research is necessary to study electron transport from CO in *G. sulfurreducens*.

The reduction of fumarate has been shown to be coupled to NAD(P)H oxidation in acetate + fumarate-grown *G. sulfurreducens*. The observed fumarate reduction rate was approximately seven times higher with NADPH compared to NADH (Galushko and Schink 2000), which is in agreement with the higher enzyme activities found in our experiments with NADP(H) compared to NAD(H) (Fig. 3). The ability to utilize NADPH for catabolism may also be inferred from the presence of an NADP^+^-dependent, but not an NAD^+^-dependent, isocitrate dehydrogenase in the TCA cycle (Galushko and Schink 2000; Methé et al. 2003). Fumarate is reduced by a bifunctional enzyme that catalyzes fumarate reduction and succinate oxidation (Butler et al. 2006). This enzyme is present at the inner side of the cytoplasmic membrane and oxidizes menaquinol for the reduction of fumarate (Galushko and Schink 2000).

The present study also showed conversion of fumarate to succinate in the absence of CO or another electron donor. The observed stoichiometry is very similar to fermentation of 7 fumarate to 6 succinate + 4 CO_2_ as performed by e.g., *Malonomonas rubra*, *Providencia rettgeri*, and *Syntrophobacter fumaroxidans* (Dehning and Schink 1989; Kröger 1974; Stams et al. 1993). However, in contrast to these bacteria, *G. sulfurreducens* did not couple fumarate conversion to growth. An explanation for this could be an impaired activity of malate oxidation reactions in the absence of acetate (Galushko and Schink 2000).

The biomass yield for growth with CO and fumarate is 0.47 mol C-biomass/mol CO compared to 0.58 mol C-biomass/mol C-acetate for growth with acetate and fumarate. The calculated Gibbs free energies of reaction (ΔG_r_) for reduction of fumarate are approximately −100 kJ/mol CO and −230 kJ/mol acetate, resulting in similar biomass yields of 4.7 mmol C-biomass/kJ for CO and 5.0 mmol C-biomass/kJ for acetate. The observations by Stouthamer (1979) of a growth yield for anaerobes of approx. 10 g dry weight/mol generated ATP and an energy requirement of 70 kJ/mol ATP equate to an energetic yield of 5.8 mmol C-biomass/kJ.

The above free energies of conversion represent approximately −50 kJ/mol electrons from CO and −30 kJ/mol electrons from acetate when coupled to fumarate reduction, which shows that energy from CO oxidation is used more efficiently for growth. For growth of *G. sulfurreducens* with acetate and fumarate, it has been shown that there is no net ATP synthesis through substrate-level phosphorylation (Galushko and Schink 2000). Energy is likely generated by electron transport phosphorylation during fumarate reduction, but the exact mechanism is not known (Galushko and Schink 2000; Butler et al. 2006). Assuming that the efficiencies of electron transport phosphorylation with electrons derived from CO and acetate are similar, an additional energy-conserving mechanism during growth with CO is needed to explain the biomass yield. A potential route to conserve energy is by electron bifurcation reactions, which thus far have not been studied for *G. sulfurreducens.* Central in electron bifurcating enzyme complexes is the presence of FAD, which can have three different potentials. Reduced flavoprotein may therefore be oxidized by two different electron acceptors with different redox potentials (Seedorf et al. 2008; Wang et al. 2010). Putative proteins with high similarity to the electron bifurcating enzyme complexes EtfAB and NfnAB in *C. kluyveri* (amino acid identity >43 %; positives >63 %) and the complex FlxABCD-HdrABC (Ramos et al. 2015) are encoded in the genome of *G. sulfurreducens*, but their role is currently unknown. The putative CO-oxidizing NADPH-reducing complex could possibly also be involved in flavin-based electron bifurcation using FAD in the FNOR subunit. Genes encoding an Rnf complex, which is important for enabling additional ATP generation in *C. kluyveri* (Seedorf et al. 2008), have not been detected in *G. sulfurreducens*.

The present study extends the range of electron donors for *G. sulfurreducens* with CO. Carbon monoxide may potentially be an important electron donor for microbial metabolism in present day anaerobic mesophilic environments, but its importance has not been very well studied. Low concentrations of CO observed in the environment, e.g., 0.2–20 nM in aquifers (Chapelle and Bradley 2007), may indicate that CO released in the degradation of organic matter is actively metabolized and therefore does not accumulate. The finding that *G. sulfurreducens* is able to use CO indicates that it may be involved in the utilization of CO in anaerobic mesophilic environments like aquifers where *Geobacter* spp. form an important part of the microbial community (Flynn et al. 2012).

The growth experiments showed tolerance of *G. sulfurreducens* to high levels of CO, which is important for potential biotechnological applications for processing of synthesis gas, which may contain up to 60 % CO (Sipma et al. 2006). With an electrode as electron acceptor, *G. sulfurreducens* efficiently converts acetate into current (Bond and Lovley 2003). If CO oxidation could be coupled to respiration with an electrode, a new technological application of *G. sulfurreducens* may be within reach, in which electrical energy is generated from synthesis gas in a microbial fuel cell (MFC). Synthesis gas consists mainly of CO and H_2_, which both can be used as electron donor by *G. sulfurreducens*. Studies with synthesis gas-fed MFCs have shown the enrichment of microbial communities consisting of acetogens, methanogens, and electricigenic microorganisms (Hussain et al. 2014). The detection of acetate in the reactor liquid suggested that CO was converted to acetate which was used as electron donor for current production (Mehta et al. 2010).

Comparative genomic analysis indicates that the capacity to use CO may be more widespread than is currently known. Gene clusters with similarity to GSU2099–2095 are present in a number of close relatives of *G. sulfurreducens* but also in more distantly related organisms (Fig. 4; Table S2). In contrast to the *Geobacter*/*Pelobacter* species which only contain putative monofunctional CODHs, the genome of *Deferrisoma camini* also encodes a putative CODH/ACS and aerobic type CO dehydrogenases (Draft genome annotations at img.jgi.doe.gov) (Table S2). The genomes of CO-utilizing *Clostridium carboxidivorans*, *Cl. autoethanogenum*, and *Cl. ljungdahlii* contain (multiple) copies of genes putatively encoding monofunctional and bifunctional CO dehydrogenases (Table S2), and the activity of the individual CODHs is not known. *Desulfotomaculum kuznetsovii* and *Dsm. nigrificans* are known to grow with CO but do not contain CooS–CooF–FNOR-encoding gene clusters. Analysis of CooS protein sequences showed that sequences that are part of highly similar gene clusters are phylogenetically also more closely related (Fig. S1). Further research is required to investigate if CO respiration without production of hydrogen as intermediate is a property that is more common in anaerobically growing microorganisms.

## Electronic supplementary material

ESM 1 (PDF 879 kb)
