# Supplementary material for: Carboxydotrophic growth of Geobacter sulfurreducens
Source: Appl Microbiol Biotechnol. 2015 Oct 19;100:997–1007. doi: 10.1007/s00253-015-7033-z (PMC4703632; doi:10.1007/s00253-015-7033-z)
Supplement: Supplementary file 1 — (PDF 879 kb) [file 253_2015_7033_MOESM1_ESM.pdf]

Supplementary Material

Applied Microbiology and Biotechnology

Carboxydotrophic growth of *Geobacter sulfurreducens*

Jeanine S. Geelhoed[a,b], Anne M. Henstra[a,*] and Alfons J.M. Stams[a]

[a]Laboratory of Microbiology, Wageningen University, Dreijenplein 10, 6703 HB Wageningen, The Netherlands
[b]NIOZ Royal Netherlands Institute for Sea Research, Korringaweg 7, 4401 NT Yerseke, The Netherlands
[*]present address: Centre for Biomolecular Sciences, University of Nottingham, University Park, NG7 2EF, Nottingham, United Kingdom

Corresponding author:
Jeanine S. Geelhoed, NIOZ Royal Netherlands Institute for Sea Research,
Korringaweg 7, 4401 NT Yerseke, The Netherlands
Phone: +31 (0)113577473
Fax: +31 (0)113573616
Email: jeanine.geelhoed@nioz.nl

Supplementary Table S1 Specific enzyme activities of cell free extracts of cultures grown with carbon monoxide, acetate or formate as electron donor.

Supplementary Table S2 Genomes containing gene clusters consisting of genes putatively encoding CooS-CooF-FNOR, denoted by locus tags of CooS indicated in bold. Also listed are locus tags for other CooS and CODH-ACS type anaerobic carbon monoxide dehydrogenase genes, and the presence of aerobic-type carbon monoxide dehydrogenase genes.

Supplementary Fig S1 Phylogenetic tree of CooS subunits of *Geobacter sulfurreducens* and known CO utilizing microorganisms and of CooS subunits of organisms not reported to use CO that have gene clusters encoding CooS, CooF and FNOR.

Supplementary Table S1

Carboxydotrophic growth of *Geobacter sulfurreducens*
Jeanine S. Geelhoed, Anne M. Henstra and Alfons J.M. Stams

Specific enzyme activities of cell free extracts of cultures grown with carbon monoxide, acetate or formate as electron donor[a]. Data for Fig. 3.

Electron donor for growth			Carbon monoxide, 40 kPa in headspace			
Cell free extract, preparation			A	B	C	D
Enzymatic conversion	E-donor	E-acceptor	Specific activity (µmol e-donor mg protein^{-1} min^{-1}) (avg ± std (n))			
CO dehydrogenase	CO, 100 kPa	BV, 2 mM	0.94 ± 0.15 (3)	3.36 ± 0.06 (2)	1.63 ± 0.01 (2)	4.09 ± 0.34 (2)
Formate dehydrogenase	Formate, 20 mM	BV, 2 mM	0.006 ± 0.0007 (3)	0.035 ± 0.008 (2)	0.005 ± 0.0001 (2)	0.005 ± 0.0016 (2)
Hydrogenase	H_2, 100 kPa	BV, 2 mM	0.033 ± 0.003 (4)	0.068 ± 0.000 (2)	0.099 ± 0.006 (3)	0.191 ± 0.012 (2)
H_2 formation	Dithionite,25mM +MV, 5 mM	protons	0.018 ± 0.0004 (3)	n.t.[b]	0.003 ± 0.0001 (2)	0.006 ± 0.0006 (3)
H_2 formation	CO, 100 kPa	protons	no activity	n.t.	no activity	no activity
NADPH oxidase	NADPH, 5 mM	BV, 2 mM	0.93 ± 0.06 (3)	1.71 ± 0.04 (2)		
NADH oxidase	NADH, 5 mM	BV, 2 mM	0.29 ± 0.04 (2)	0.50 ± 0.001 (2)		
	NADH, 20 mM	BV, 2 mM	0.34 ± 0.02 (2)	0.40 ± 0.04 (4)		
NADPH formation	CO, 100 kPa	$NADP^+$, 5 mM	0.062 ± 0.004 (2)	0.15 ± 0.02 (2)		
NADH formation	CO, 100 kPa	NAD^+, 5 mM	0.013 ± 0.008 (3)	0.005 ± 0.003 (2)		

[a] Fumarate (40 mM) as electron acceptor

[b] n.t. = not tested

(continued). Specific enzyme activities of cell free extracts of cultures grown with carbon monoxide, acetate or formate as electron donor[a]

Electron donor for growth				Acetate, 16 mM		Formate, 40 mM[c]
Cell free extract, preparation			E	F	G	H
Enzymatic conversion	E-donor	E-acceptor	Specific activity (µmol e-donor mg protein^{-1} min^{-1}) (avg ± std (n))			
CO dehydrogenase	CO, 100 kPa	BV, 2 mM	0.21 ± 0.01 (2)	0.076 ± 0.005 (2)	0.30 ± 0.06 (4)	5.96 ± 0.21 (3)
Formate dehydrogenase	Formate, 20 mM	BV, 2 mM	0.64 ± 0.009 (2)	0.40 ± 0.02 (2)	1.57 ± 0.17 (6)	1.80 ± 0.17 (3)
Hydrogenase	H_2, 100 kPa	BV, 2 mM	1.04 ± 0.09 (2)	0.74 ± 0.03 (2)	1.65 ± 0.06 (3)	0.53 ± 0.033 (3)
H_2 formation	Dithionite,25mM +MV, 5 mM	protons	n.t.	n.t.	0.20 ± 0.017 (3)	0.24 ± 0.023 (3)
H_2 formation	CO, 100 kPa	protons	n.t.	n.t.	n.t.	n.t.
NADPH oxidase	NADPH, 5 mM	BV, 2 mM	1.54 ± 0.02 (2)	1.35 ± 0.1 (2)		
NADH oxidase	NADH, 5 mM	BV, 2 mM	0.20 ± 0.01 (2)	0.062 ± 0.002 (2)		
	NADH, 20 mM	BV, 2 mM	0.31 ± 0.01 (2)	0.082 ± 0.004 (2)		
NADPH formation	CO, 100 kPa	$NADP^+$, 5 mM	n.t.	n.t.		
NADH formation	CO, 100 kPa	NAD^+, 5 mM	n.t.	n.t.		

[a] Fumarate (40 mM) as electron acceptor

[b] n.t. = not tested

[c] 0.8 mM acetate added

Supplementary Table S2

Carboxydotrophic growth of *Geobacter sulfurreducens*

Jeanine S. Geelhoed, Anne M. Henstra and Alfons J.M. Stams

Genomes containing gene clusters consisting of genes putatively encoding CooS-CooF-FNOR, denoted by locus tags of CooS indicated in bold. Also listed are locus tags for other CooS and CODH-ACS type anaerobic carbon monoxide dehydrogenase genes, and the presence of aerobic-type carbon monoxide dehydrogenase genes.

Species	Growth with CO	CooS	CODH-ACS	Aerobic-type CO dehydrogenase
Geobacter sulfurreducens	+	GSU**2098**		
Geobacter daltonii	n.r.[a]	Geob_0362, **1046**		
Geobacter uraniireducens	n.r.	Gura_**0618**		
Pelobacter carbinolicus	n.r.	Pcar_**0057**		
Deferrisoma camini	n.r.	DefcaDRAFT_3102[b], **3435**	DefcaDRAFT_2710	3 aerobic-type
Clostridium carboxidivorans	+	CcarbDRAFT_0341, **1756**	CcarbDRAFT_0164, 2944	
Clostridium autoethanogenum	+	CAETHG_**3005**, 3899	CAETHG_1621/1620	
Clostridium ljungdahlii	+	CLJU_**c09110**, c17910	CLJU_c37670	
Desulfotomaculum acetoxidans	n.r.	Dtox_2327, **2933**	Dtox_1270, 3780	
Desulfotomaculum gibsoniae	n.r.	Desgi_2753, **3080**	Desgi_2052	≥6 aerobic-type
Caldicellulosiruptor saccharolyticus	n.r.	Csac_**0400**		
Desulfobacterium autotrophicum	n.r.	HRM2_**43440**		
Desulfobacula toluolica	n.r.	TOL2_00990, 38110		
Azotobacter vinelandii	n.r.	Avin_04490		
Carboxydothermus hydrogenoformans	+	CHY_0034, 0085, 0736, 1824	CHY_1221	
Carboxydothermus ferrireducens	+	CarfeDRAFT_00002400, 00009660, 00010430	CarfeDRAFT_00014810	1 aerobic-type

[a]n.r.= not reported. [b]Grey shading indicates the presence of a gene cluster with a different order of genes putatively encoding CooS, CooF and FNOR.

Supplementary Fig S1

Carboxydotrophic growth of *Geobacter sulfurreducens*

Jeanine S. Geelhoed, Anne M. Henstra and Alfons J.M. Stams

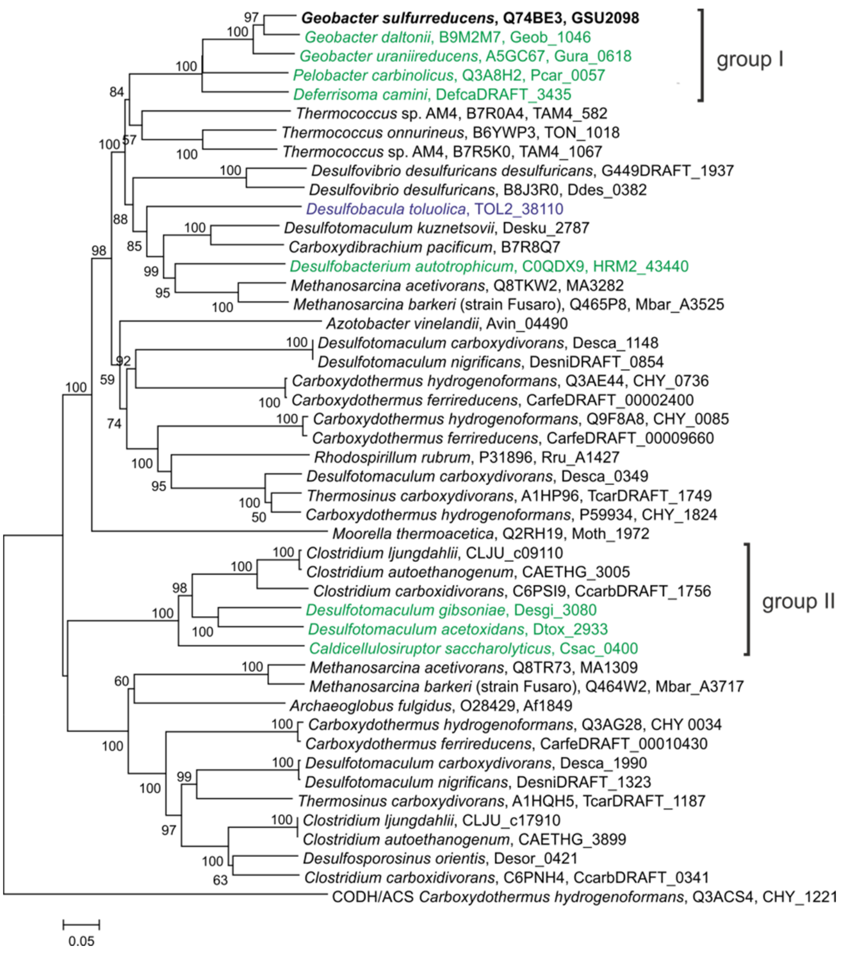

Phylogenetic tree of CooS subunits of *Geobacter sulfurreducens* (bold) and known CO utilizing microorganisms (black) and of CooS subunits of organisms not reported to use CO that have gene clusters encoding CooS, CooF and FNOR (in this order: green, see also Fig. 4, or in a different order: blue, see also Table S2). The CODH subunit of the CO dehydrogenase/acetyl-CoA synthase complex of *C. hydrogenoformans* was used as outgroup. Accession numbers in UniProt and/or Locus tags in IMG are listed. Bootstrap values ≥ 50% are indicated at the nodes. The scale bar indicates the evolutionary distance corresponding to 5 substitutions per 100 amino acids.

Phylogenetic analysis of monofunctional CODH (CooS) protein sequences showed that CooS sequences of *Geobacter*/*Pelobacter* sp. and *Deferrisoma camini* are placed together (Group I). The proteins in Group I are most closely related to CooS proteins of the Archaeal *Thermococcus* spp., that oxidize CO + H_2O to H_2. The deltaproteobacterial CooS proteins of the CO-oxidizing sulfate reducing bacterium *Desulfovibrio desulfuricans* are more distantly related. The CooS protein sequences of *Clostridium carboxidivorans*, *Cl. autoethanogenum* and *Cl. ljungdahlii* that are part of CooS-CooF-FNOR gene clusters grouped together with the CooS protein sequences of *Caldicellulosiruptor saccharolyticus* and *Desulfotomaculum acetoxidans* and *Dsm. gibsoniae* that are also part of such clusters (Group II). The CooS protein sequences of *Desulfobacterium autotrophicum*, *Carboxydothermus hydrogenoformans* (CHY0736), *Desulfobacula toluolica* and *Azotobacter vinelandii* are scattered over the tree and not closely related to other putative CooS proteins that can be directly linked to CooF and FNOR.
